# The Role of Acupuncture in Hormonal Shock-Induced Cognitive-Related Symptoms in Perimenopausal Depression: A Randomized Clinical Controlled Trial

**DOI:** 10.3389/fpsyt.2021.772523

**Published:** 2022-01-13

**Authors:** Jun-He Zhou, De-Long Zhang, Bai-Le Ning, Xiao-Juan Xue, Lin Zhao, Qian Wu, Lu-Da Yan, Ming Liu, Wen-Bin Fu

**Affiliations:** ^1^Key Laboratory of Brain, Cognition and Education Sciences, Ministry of Education, Guangzhou, China; School of Psychology Center for Studies of Psychological Application, and Guangdong Key Laboratory of Mental Health and Cognitive Science, South China Normal University, Guangzhou, China; ^2^The Second Clinical College, Guangzhou University of Chinese Medicine, Guangzhou, China; ^3^Shenzhen Bao'an Traditional Chinese Medicine Hospital, Guangzhou University of Chinese Medicine, Guangzhou, China; ^4^Innovative Research Team of Acupuncture for Depression and Related Disorders, The Second Affiliated Hospital of Guangzhou University of Chinese Medicine (Guangdong Provincial Hospital of Chinese Medicine), Guangzhou, China

**Keywords:** perimenopause, depression, acupuncture, embodied cognition, QOL (quality of life)

## Abstract

**Introduction:** Perimenopausal depression is predominantly caused by hormone shock, but the underlying physical and psychological factors are still unclear.

**Objectives:** To explore the constituent components of perimenopausal depression by dynamically depicting its influencing factors and interactive pathways from the perspective of embodied cognition.

**Methods:** This is a randomized clinical controlled trial. In this study, electroacupuncture was compared with escitalopram. A total of 242 participants with mild to moderate perimenopausal depression were enrolled from 6 hospitals in China. Each participant had a 12-week intervention and a 12-week follow-up period. The primary outcome of this study is the Hamilton Depression Rating Scale (HAMD-17), and the secondary outcome is the menopause-specific quality of life scale (MENQOL), serum Follicle-stimulating hormone (FSH), luteinizing hormone (LH), and estrogen (E_2_) levels.

**Results:** The structural equation model suggested that hormone levels were not directly associated with HAMD-17 (*P* = 0.852), while MENQOL was statistically correlated with HAMD-17 as an intermediary variable (*P* < 0.001). Electroacupuncture gradually showed positive impacts on MENQOL and HAMD-17 during the follow-up period (*P* < 0.05). Cognitive impairment is the dominant dimension of perimenopausal depression.

**Conclusions:** Hormonal shock may affect clinical symptoms and poor quality of life to induce cognitive impairment leading perimenopausal depression, and this impact on cognition is embodied. Electroacupuncture has positive effect on perimenopausal depression and quality of life.

## Introduction

Depression is one of the most typical psychosomatic diseases in modern society and is characterized by negative mood, delayed thinking, cognitive impairment, and physical symptoms (1). Perimenopausal depression in women, a type of depression, is caused by substantial estrogen shock and provides an avenue to explore psychosomatic factors and their relationships (2). It is generally believed that both physical and psychological changes can cause depression (3). However, the psychological and physiological interactions of perimenopausal depression remain unclear.

Eighty percent of women tend to develop perimenopause at an average age of ~51.4 years. Although perimenopause is usually considered a reproductive transition, its clinical symptoms can include measurable neurological symptoms, such as insomnia, depression, and memory impairments (4). Longitudinal studies have found that the risk ratio of depression during perimenopause is as high as 1.8–2.9 (5). Estrogen activates GTPase-driven signaling cascades through G protein coupled estrogen receptor (GPER) and activates the cyclic adenosine monophosphate (cAMP), phosphatidylinositol-3-kinase (PI3K)-Akt and extracellular-signal-regulated kinases (MAPK-Erk) signaling pathways, which are involved in learning, memory, synaptic transmission and growth through brain-derived neurotrophic factor (BDNF) (6–11). Using PET, we showed that patients had lower pons metabolism and higher metabolism in the middle and inferior (Broca's) frontal gyrus (12) compared to women without menopausal depression. In perimenopausal women with cognitive impairment, one prior PET study showed glucose metabolic decline (13) in the hippocampus, parahippocampal gyrus, temporal lobe, medial prefrontal cortex, and posterior cingulate gyrus. Additionally, some studies have demonstrated that estrogen receptors are widely distributed in the hypothalamus, amygdala, hippocampus, and cingulate cortex (14).

Embodied cognition provides a new perspective to re-understand the interaction of psychosomatic factors in menopausal depression. Cognitive impairment is a clinical symptom of people in perimenopause, which is reflected in memory, language, learning and other abilities (15) and is also a major manifestation of depression (16). Moreover, hormonal shock, negative emotions, and reduced quality of life can also affect cognitive function (17–20), and this cognitive influence belongs to the category of embodied cognition. Embodied cognition believes that the environment and one's somatosensory experience will affect cognitive function (21). Electroacupuncture increases the patient's physical sensations. Thus, to explore the regulatory effect of somatic electrical stimulation on the psychosomatic factor pathway of depression, we applied electroacupuncture to increase the patient's physical sensations. This increase in sensations is also a way to affect embodied cognition.

To deepen the relationship between an embodied cognitive framework and psychosomatic factors in patients with menopausal depression, this study combined structural equation and potential profile analysis methods to investigate the roles of psychosomatic factors. Furthermore, we conducted a correlation analysis of hormones, quality of life, symptoms, and depression in patients with perimenopausal depression and constructed structural equations to explore the relationships between them. Based on the structural equation model, we used a randomized controlled trial to observe the differences between electroacupuncture and escitalopram in improving the quality of life, perimenopausal, and depressive symptoms in patients with perimenopausal depression. A randomized controlled study was established to evaluate differences in the efficacy of electroacupuncture and antidepressants in patients with mild to moderate depression during perimenopause and to evaluate whether the efficacy of antidepressants is dominated by improving cognitive impairment.

## Methods

### Trial Design

This is a parallel randomized controlled trial. Using central randomization system, and the ratio of participants in the two groups is 1:1. We use the stratified randomization method, the center was used as a stratification factor, and participants were randomly assigned to the electroacupuncture group and the medication group according to a ratio of 1:1. The “Proc plan” program of SAS9.3 statistical analysis software was used to generate the random plan needed for the study. The random serial number and random plan are in charge of the Clinical Evaluation Center of the Clinical Institute of China Academy of Chinese Medical Sciences. When the researchers registered the information of participant who meet the inclusion criteria into the central randomization system, the system will generate a random serial number and distribute the participant into a group. Researchers from each hospital arrange participants for treatment according to random results.

### Sample Size

The study adopted a non-inferiority trial design. According to previous studies (22, 23), the reduction rate of drug treatment for perimenopausal depression or symptoms is about 80%, we set the effective rate of the two groups to be 0.8. The test level is set to α = 0.05, β = 0.10, and the δ value is 0.16 (not exceeding 1/5 of the medication group). Calculate the sample size of both groups are 107 participants. Taking into account the 15% dropout rate, it was calculated that a total of 252 participants were needed. The study actually recruited 252 participants, excluding 10 participants who did not meet the criteria and refused to participate in this study, and a total of 242 participants were randomly divided into an acupuncture group (EA) and a medication group (MC).

### Blinding

This study is not a double-blind trial. Because of different intervention methods, blinding between doctors and patients cannot be achieved.

### Participants

A total of 327 patients were enrolled in this study. Among the enrolled patients, a total of 75 patients did not meet the inclusion or exclusion criteria after diagnosis and screening. Finally, 252 participants were recruited into this study. After re-assessment by gynecologists and psychiatrists, 8 participants did not meet the diagnostic criteria for perimenopausal depression, and 2 participants withdrew from the study. Therefore, 242 participants from 6 different hospitals in China participated in this study. The participants all had depressive disorders, were in the perimenopausal period and had not experienced depressive disorders in the past. The participants enrolled met the perimenopausal diagnosis criteria updated by the American Society of Reproductive Society (STRAW-10) in 2012 and the American Psychiatric Association DSM-5 criteria of Depressive Disorder. The HAMD-17 score of all enrolled patients ranged from 8 to 23, and the depression diagnosis was confirmed by a psychiatrist to meet the criteria for the mild to moderate depression group.

### Inclusion Criteria

Perimenopausal depression participants (aged 45–55 years, 8 ≤ HAMD < 23) were eligible for enrollment if they conformed to the criteria in STRAW-10 and DSM-5 and had not used any hormone replacement therapy or antidepressant drugs during the last 3 months. The participants were experiencing first-onset depressive disorder while agreeing to participate in the study and signing the informed consent form.

### Exclusion Criteria

Exclusion criteria included allergy to escitalopram and other drugs that may cause drug-to-drug interactions. Patients with ovarian cysts, uterine fibroids larger than 4 cm in diameter, ovaries or after hysterectomy, cognition disorder, SCL-90 score failed to rule out suicidal tendencies (> 26), and diseases considered by the investigator to be inappropriate for participating in this study will be excluded.

### Interventions

The EA procedure adhered to the Standards for Reporting Interventions in Clinical Trials of Acupuncture (STRICTA) standard. The acupuncture points in the EA group were BAIHUI (DU20), YINTANG (EX-NH3), GUANYUAN (RN4), ZIGONG (EX-CA1, bilateral), TIANSHU (ST25, bilateral), HEGU (L14, bilateral), TAICHONG (LR3, bilateral), and SANYINJIAO (SP6, bilateral). TIANSHU uses a 3-CUN tube needle, ZIGONG and GUANYUAN use a 2-CUN needle. The depth of acupuncture should reach the peritoneal wall, and the patient's feeling of local acupuncture pain and resistance under the acupuncturist's hand as the standard. The acupuncturist only pierces the TIANSHU and ZIGONG acupoints without lifting and twisting. GUANYUAN is evenly lifted and twisted 3 times in a small range, and the participant feels soreness in the area of GUANYUAN. HEGU, TAICHONG, and SANYINJIAO use a 1.5-CUN tube needle and acupuncture to a depth of 0.8 to 1 CUN, lifting and twisting 3 times. BAIHUI and YINTANG use a 1-CUN tube needle, which is pierced to a depth of 0.5-CUN at an angle of 30 degrees to the rear, and is lifted and twisted 3 times, with the degree of local soreness. Participants in the acupuncture group underwent a connected 50 Hz, 0.5–10 mA current sparse and density wave stimulation between the needle handles of BAIHUI and YINTANG, bilateral ZIGONG and bilateral TIANSHU, and each treatment occurred three times per week for 30 min, with an interval of more than 24 h. All acupuncture operations are performed by acupuncturists with more than 5 years of clinical experience. In the MC group, 10 mg of escitalopram was taken orally after breakfast every day for 12 weeks. If the participant has an adverse reaction due to the escitalopram, the escitalopram dose can be halved to 5 mg per day. The study period for all participants was 24 weeks, the treatment intervention period after enrollment was 12 weeks, and the follow-up period was 13–24 weeks.

### Measures

This study uses HAMD-17 as the primary outcome, which has five dimensions: somatization, weight, cognitive impairment, block, and sleep. HAMD-17 score reduction rate ≥50% is significantly effective, ≥ 25%, < 50% is effective, and <25% is invalid. Assessment of the HAMD-17 scale was performed at 0, 4, 8, 12, 16, and 24 weeks. The perimenopausal quality of life of participants was evaluated by the MENQOL scale, which has four dimensions: vasodilation, psychological symptoms, physical symptoms, and sexual life. The MENQOL scale was measured at 0, 4, 8, 12, 16, 20, and 24 weeks. At weeks 0 and 12, venous blood was drawn to quantify the participant's hormone levels, including follicle-stimulating hormone (FSH), estradiol (E_2_), and luteinizing hormone (LH). Hormones were collected on days 2–5 during the menstrual cycle in weeks 0 and 12 of the treatment period, and FSH, LH, and E_2_ were taken from venous blood drawn at 8–9 A.M. on an empty stomach. This study will record the adverse events of the participants during the study and evaluate whether the adverse reactions are related to the intervention. Researchers will make corresponding treatment for adverse events and record the final prognosis. Adverse events related to acupuncture in the study included pain, dizziness, hematoma, and infection. Adverse reactions related to medication included gastrointestinal reactions, dizziness, allergies, and mood disorders.

### Statistical Analysis

Unless otherwise specified, an intent-to-treat (ITT) analysis was performed. ITT included all patients who participated in at least one treatment, including participants who dropped out. All statistical analyses were performed using SPSS (IBM SPSS Statistics version 24.0) and MPLUS (version 8.0). The statistical analysis was divided into three procedures: baseline analysis, construction of a structural equation model, and latent profile analysis (LPA) of the HAMD-17.

### Structural Equation Model

The variables FSH, LH, and E_2_ were defined as latent variables for hormones, the 5 dimensions of the HAMD-17 were defined as latent variables for depression, and the 4 dimensions of MENQOL were defined as latent variables for perimenopausal symptoms and quality of life. Pearson and Spearman correlation analyses were used to test the relationship between the dimensions of latent variables. We used a structural equations model (SEM) to test the mediation relationship among latent variables. The structural equation model was conducted by using MPLUS (version 8.0). The model fit was assessed with the comparative fit index (CFI, critical value ≥ 0.9), the Tucker Lewis Index (TLI, critical value ≥ 0.9), the root mean square error approximation (RMSEA, value blew 0.08), and the chi-square divided by degrees of freedom < 3 (χ^2/df^).

### Baseline Analysis

First, the independent sample *t*-test was used to compare the significant differences between normality and homogeneity. The Mann-Whitney U test was selected for those that did not meet the criteria for normality and homogeneity. We further used the chi-square test or Fisher's exact test to analyze the categorical dataset. For comparison of ordered classification data, we used the rank sum test.

### Efficacy Evaluation Between Two Groups

The differences between weeks 4, 8, 12, 16, 20, and 24 and week 0 were taken as the changes in efficacy. Repeated measures analysis of variance was used to compare the curative effect between the two groups. If there is an interaction between time and group, the two sets of data at each time point will be compared separately. For data conforming to normality and homogeneity of variance, an independent sample *t*-test was used, and the Mann-Whitney U test was used for data that did not meet the above criteria. The data are represented by the mean ± SD. A *P* < 0.05 was considered significantly different.

### Latent Profile Analysis

Latent profile analysis was used to distinguish the characteristics of different dimensions on the HAMD-17 scale. The MPLUS could complete the LPA in HAMD-17 scale. Each latent profile analysis was analyzed from a 2-class model, and the number of classes was gradually increased until the optimal model was fitted. Model fit was assessed from three indices: information criteria (CI), entropy and Lo-Mendell-Rubin (LMR). Information criteria consisted of the Akaike information criterion (AIC), Bayesian information criterion (BIC) and adjusted Bayesian information criterion (aBIC). A better model fit included the smallest information criteria, entropy approaching 1, and Lo-Mendell-Rubin *P* < 0.05.

## Results

### Structural Equation Model of Hormones, MENQOL and HAMD

**Figure 1:**
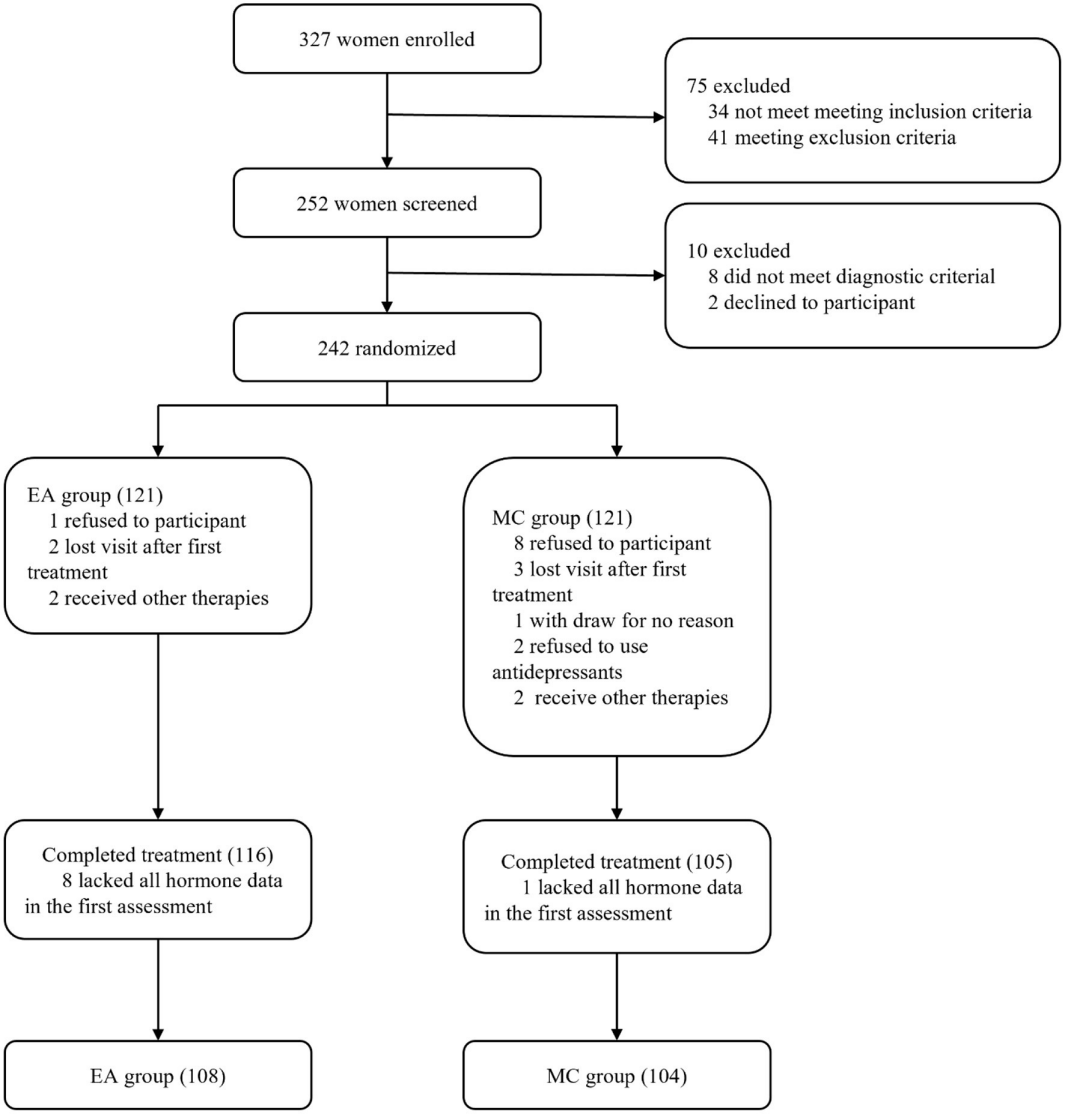
Research flow chart. EA represents the electroacupuncture group, and MC represents the escitalopram group.

**Figure 2:**
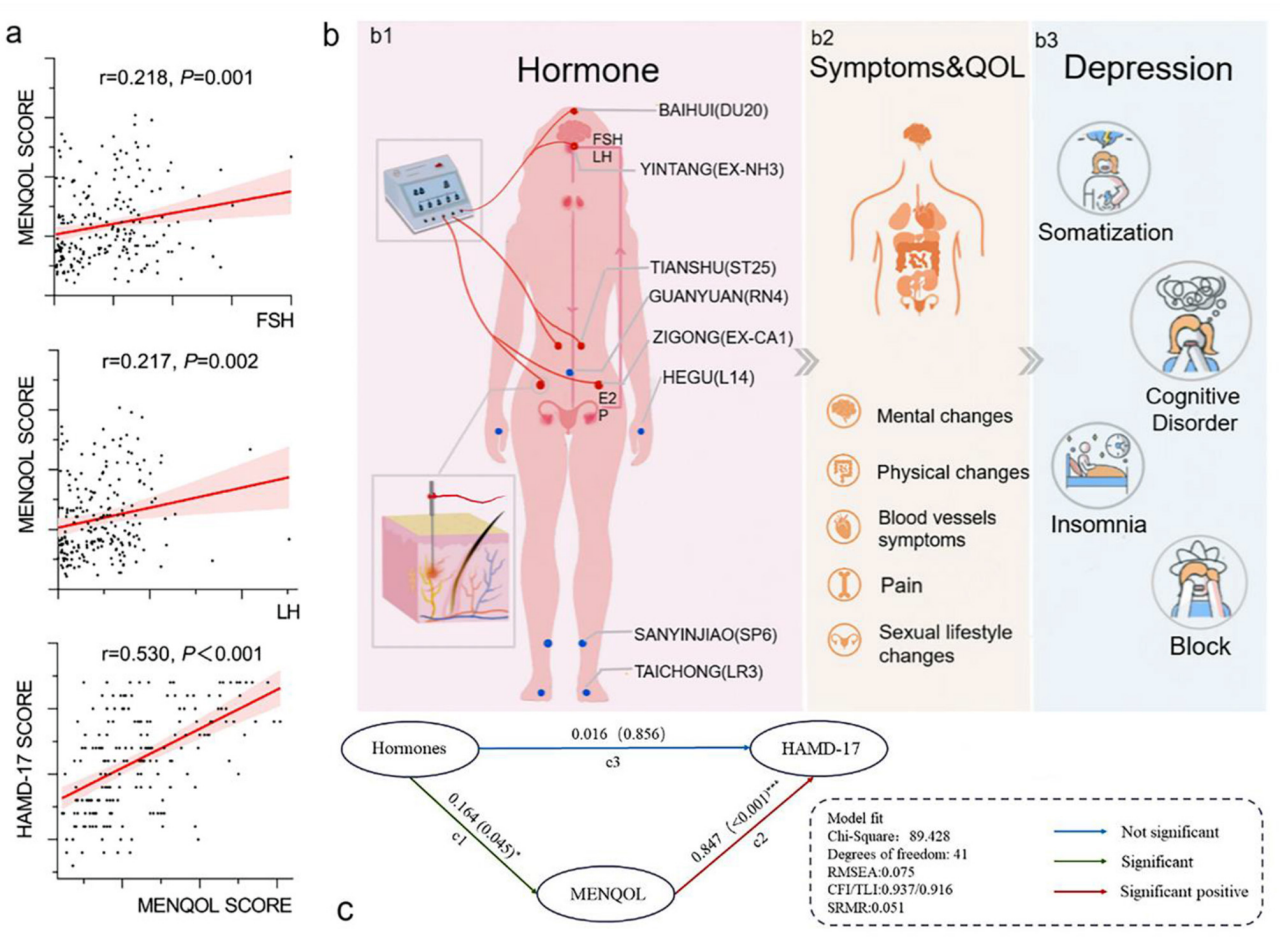
Correlation analysis and structural equation model. **(a)** Both FSH and LH have a positive correlation with MENQOL, and MENQOL has a positive correlation with HAMD. (b1) Each wire of the electroacupuncture device connects 2 acupoints to form a loop, and the wire of the electroacupuncture device is connected to the needle handle. Hormones, including FSH, LH, E2, and hormone shock, are the main factors associated with a series of symptoms of perimenopause. (b2) Symptoms and quality of life include common clinical symptoms of perimenopausal patients, most of which are caused by hormone shock. (b3) Symptoms and quality of life are related to depression, and cognitive impairment is the dominant dimension in depressive symptoms. **(c)** Structural equation model of mediation effect. Structural equation model of mediation effect. The latent variable hormones include FSH and LH. MENQOL is the intermediary variable of the model. Hormones point to MENQOL, and MENQOL points to HAMD-17. The relationship between hormones, symptoms and quality of life and depression.

A total of 242 participants were included in the study, and they were randomly assigned to the EA group and the MC group. Among all participants, 221 participants completed the treatment, including 116 in the EA group and 105 in the MC group. Nine participants were excluded because of a lack of all hormone data in the first assessment. The final number of participants was 212, including 108 in the EA group and 104 in the MC group. The research flow chart is shown in Figure 1. The correlation analysis among the three variables (hormones, MENQOL, and HAMD-17) of 212 participants before treatment is shown in Figure 2. Because not all dimensional variables fit the normal distribution, Spearman correlation analysis was used for the correlation analysis. The results of the correlation analysis showed that there was a significant association between HAMD-17 and MENQOL. For the hormones, FSH (*r* = 0.218, *P* = 0.001) and LH (*r* = 0.217, *P* = 0.002) were all correlated with MENQOL scales, but estradiol had no significant correlation with MENQOL and HAMD-17. Finally, the equation was constructed with hormones (FSH and LH), MENQOL, and HAMD-17, where MENQOL showed a significant mediation variable (Figure 2). The results indicated that MENQOL was statistically significant as a mediating variable between hormones (*r* = 0.164, *P* = 0.045, [95% CI, 0.010–0.317]) and HAMD-17 (*r* = 0.847, *P* < 0.001, [95% CI, 0.670–1.016]), while the direct path between hormones and HAMD-17 was not significant (*r* = 0.016, *P* = 0.856, [95% CI,−0.151 to 0.191]).

### Baseline and Efficacy Data

**Figure 3:**
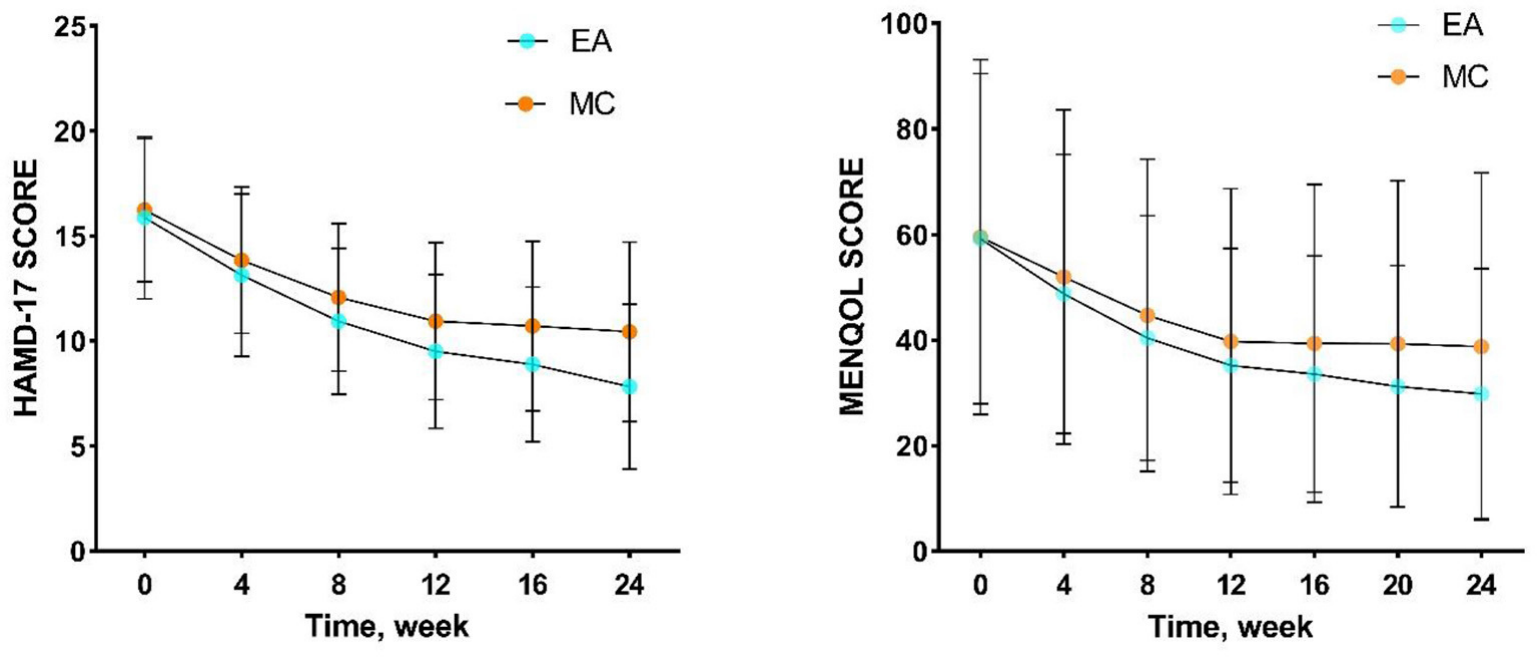
Trends in the efficacy of HAMD-17 and MENQOL. EA, acupuncture group; MC, medication group.

In baseline, the comparison between the two groups showed no significant difference in terms of race, marriage, occupational status, BMI, perimenopausal staging, liver function, kidney function, hormones, HAMD-17, and MENQOL (Table 1). The results of repeated measure data analysis are as follows. For HAMD-17, the data does not satisfy the Mauchly 's Test of Sphericity (*P* < 0.01). We use the Greenhouse-Geisser method to test, and the results show the time factor (*F* = 250.256, *P* < 0.01), time^*^group (*F* = 5.893, *P* = 0.001), and group factor (*F* = 10.435, *P* = 0.001). For MENQOL, the data does not satisfy the Mauchly 's Test of Sphericity (*P* < 0.01). We use the Greenhouse-Geisser method to test, and the results show the time factor (*F* = 209.351, *P* < 0.01), time^*^group (*F* = 5.130, *P* = 0.009), and group factor (*F* = 1.889, *P* = 0.171). The change trend of curative effect is shown in Figure 3. For the comparison at different time points, the results indicated that the MENQOL and HAMD-17 scores between the two groups showed a downward trend. Within 12 weeks of intervention, there was no difference between the two groups in terms of hormones, MENQOL, or HAMD-17 (*P* > 0.05). During the follow-up period, the EA group showed a significant difference between the MC group in MENQOL (week 20, *P* = 0.011, week 24, *P* = 0.008) and HAMD-17 (week 16, *P* = 0.023, week 24, *P* = 0.001) (Table 2).

**Table 1:** Baseline data.

**Variable**	**Category**	**EA (*N* = 108)**	**MC (*N* = 104)**	***P***
Race	Han	101 (93.5%)	100 (96.2%)	0.539
	Others	7 (6.5%)	4 (3.8%)	/
Marital status	Married	1 (0.9%)	0 (0%)	0.239
	Unmarried	107 (99.1%)	102 (98.1%)	/
	Divorced	0 (0%)	2 (1.9%)	/
Working status	Employed	63 (58.3%)	56 (52.9%)	0.490
	Unemployed	45 (41.7%)	49 (47.1%)	/
BMI	/	22.65 ± 2.60	22.40 ± 2.44	0.482
Stage	Early menopausal transition	45 (41.7%)	41 (39.4%)	0.839
	Late menopausal transition	24 (22.2%)	27 (26.0%)	/
	Early postmenopause	39 (36.1%)	36 (34.6%)	/
ALT (U/L)	/	20.44 ± 9.05	19.73 ± 10.17	0.347
AST (U/L)	/	22.49 ± 7.92	22.53 ± 6.58	0.764
TBIL (μmol/L)	/	12.18 ± 5.25	12.46 ± 5.84	0.853
BUN (mmol/L)	/	5.17 ± 4.98	4.87 ± 5.00	0.064
Cr (μmol/L)	/	60.98 ± 18.49	58.94 ± 20.14	0.218

*BMI, body mass index; ALT, alanine transaminase; AST, glutamic oxalacetic transaminase; TBIL, total bilirubin; BUN, urea nitrogen; Cr, creatinine*.

**Table 2:** Efficacy data.

**Week**	**Variable**	**EA (N = 108)**	**MC (I = 104)**	**P**
Week 0	FSH (pmol/L)	42.24 ± 34.63	45.86 ± 35.65	0.400
	E_2_ (IU/L)	179.52 ± 250.88	146.02 ± 203.22	0.287
	LH (IU/L)	22.91 ± 17.35	23.90 ± 19.07	0.891
Week 12	FSH (pmol/L)	44.04 ± 35.65	44.32 ± 35.78	0.409
	E2 (IU/L)	156.97 ± 250.88	169.16 ± 267.11	0.241
	LH (IU/L)	23.58 ± 18.61	23.82 ± 17.94	0.574
**Week** **n** **- Week 0 (change from Week 0)**				
Week 0	MENQOL	60.65 ± 32.13	60.32 ± 34.48	0.734
Week 4 -Week 0		11.48 ± 13.28	7.68 ± 8.26	0.209
Week 8 - Week 0		19.60 ± 18.56	14.65 ± 13.69	0.199
Week 12 - Week 0		25.15 ± 22.74	19.60 ± 17.13	0.348
Week 16 - Week 0		26.50 ± 22.73	20.01 ± 18.82	0.088
Week 20 - Week 0		28.45 ± 22.76	19.93 ± 19.85	0.011^*^
Week 24 - Week 0		29.77 ± 23.92	21.07 ± 20.74	0.008^**^
**Week** **n** **- Week 0 (change from Week 0)**				
Week 0	HAMD-17	16.06 ± 3.90	16.09 ± 3.48	0.518
Week 4 - Week 0		2.90 ± 2.95	2.41 ± 2.46	0.467
Week 8 - Week 0		5.02 ± 3.36	4.07 ± 3.53	0.064
Week 12 - Week 0		6.45 ± 4.51	5.12 ± 3.86	0.052
Week 16 - Week 0		6.94 ± 4.56	5.40 ± 4.00	0.023^*^
Week 24 - Week 0		7.81 ± 4.82	5.76 ± 4.16	0.001^**^

*FSH, follicle-stimulating hormone; E_2_, estrogen; LH, luteinizing hormone; MENQOL, Menopause-Specific Quality of Life Questionnaire; HAMD, Hamilton Depression Scale*.

** *P < 0.01*,

* *P < 0.05*.

### Latent Profile Analysis of HAMD-17 at Different Times

**Figure 4:**
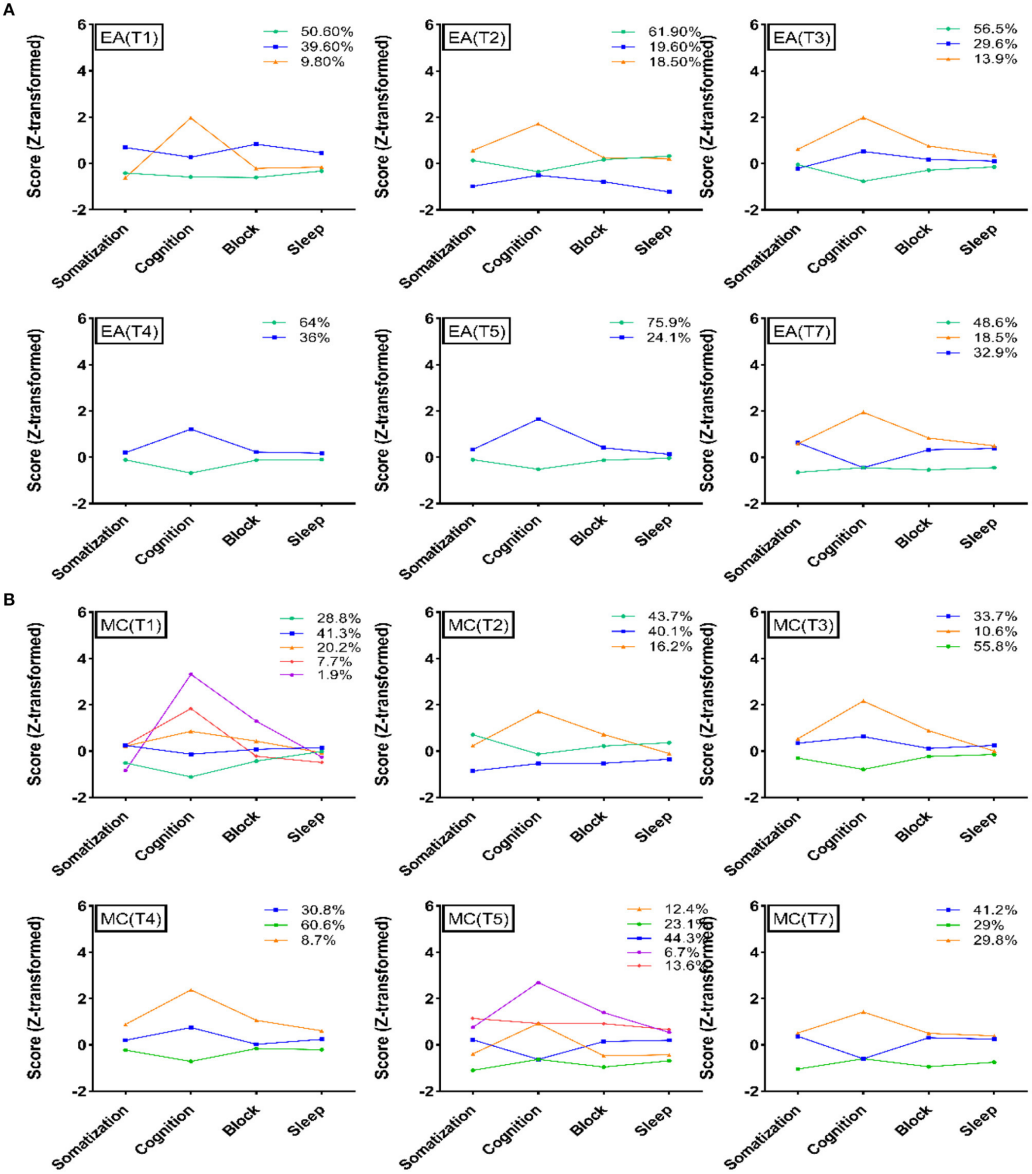
Latent profile analysis of the two groups. **(A)** Latent profile analysis of HAMD-17 at different evaluation time points in the electroacupuncture group. **(B)** Latent profile analysis of HAMD-17 at different evaluation time points in the escitalopram group. EA, acupuncture group; MA, medication group; T1, 0 week; T2, 4 weeks; T3, 8 weeks; T4, 12 weeks; T5, 16 weeks; T7, 24 weeks.

First, all datasets were transformed to Z-values before the LPA. Considering that most participants in the 3 categories with weighted scores had normal scores, they were not included in the LPA analysis. According to the results, a 4-class was the expected classification in the LPA of HAMD-17. Second, we analyzed the HAMD-17 scores of EA and MC participants during different periods of treatment to observe the trends of the participants in different dimensions. All LPA results showed that the depression patients were mainly classified according to the dimension score, and the participants with a high HAMD-17 total score had relatively high scores in each dimension. Among the four dimensions, cognitive impairment was the main dimension that dominated the differences in group classification. In the treatment process for the two interventions, the proportion of HAMD-17 low score classification gradually increased, and cognitive impairment was still the main distinguishing dimension of patient classification. For more details, please refer to Figure 4.

### Safety Outcomes

The adverse events in the study were mild to moderate events, some of which were related to acupuncture and drug side effects. The overall frequency of adverse events in this study is relatively low, suggesting that acupuncture and drugs have a certain degree of safety in the treatment of perimenopausal depression, but it is still necessary to pay attention to the changes in patients' symptoms. The summary of adverse events is shown in Table 3.

**Table 3:** Adverse reactions.

**Group**	**Symptoms**	**Number of cases**	**Relevant/irrelevant**	**Treatment**	**Prognosis**
EA	Pain	4	0/4	No special treatment	Restoration
	Dizziness	3	3/0	Rest	Restoration
	Hematoma	4	4/0	Hemostasis	Restoration
	Infection	0	0	/	/
	Others	0	0	/	/
MC	Dizziness	6	0/6	Rest	Restoration
	Gastrointestinal reaction	5	5/0	Reduce the dose and rest	Restoration
	Allergy	0	0	/	/
	Mood disorders	0	0	/	/
	Exhausted	1	1/0	Rest	Restoration
	Others	0	0	/	/

*EA, acupuncture group; MC, medication group*.

## Discussion

This study focuses on the premenopausal depression population. From the perspective of embodied cognition, we constructed a structural equation between hormones, symptoms, and depression and used LPA to analyze the dynamic changes in the curative effect of electroacupuncture and escitalopram. We found that perimenopausal depression hormone shock induces cognitive-related symptoms, which further lead to cognitive impairment. Cognitive impairment is the leading factor in perimenopausal depression and ultimately dominates depression. As a kind of electrical stimulation with strong somatosensory properties, electroacupuncture has the effect of improving perimenopausal depression.

Perimenopause is a special physiological period for women. Depression easily occurs during this period (24, 25). Perimenopausal depression is different from general depression (26–28) and is caused by hormonal shock (29, 30). The fluctuation of FSH and LH leads to changes in women's hot flashes, bones, vascular endothelium, arteriosclerosis, and lipid metabolism (31), and women in perimenopause are more likely to have physical symptoms and poor quality of life. Although depression is one of the clinical symptoms in perimenopausal women, they do not always suffer from depression. However, women with obvious hormone shock and strong hormone sensitivity are more vulnerable to depression (18, 19). The correlation between depression and physical symptoms has been confirmed, and depression is easily affected by self-discomfort symptoms (32). In this study, we used MENQOL as a mediating variable to connect hormones and HAMD-17, and the model fit was well-established. The model suggested that hormones (both FSH and LH) were associated with MENQOL and that MENQOL was correlated with depression (HAMD-17), but hormones were not directly related to depression. It seems that hormone shock may not directly predict the occurrence and severity of depression, but perimenopausal symptoms and quality of life can be used as intermediate variables for the path from hormones to depression. We have not seen the correlation between estrogen and MENQOL from our data. However, in previous report (31), it has been repeatedly mentioned that the fluctuation of estrogen in the perimenopausal period will cause patients to have symptoms such as hot flashes, irritability, mood disorders, and insomnia. This is inconsistent with the results of our research. The reason why the correlation between estrogen and MENQOL is not obvious may be due to insufficient sample size and insufficient number of hormone tests. There is no correlation between hormones and HAMD-17 in our data. It may be that fluctuations in hormones make it easier for perimenopausal women to have perimenopausal symptoms, such as hot flashes. The obvious response to perimenopausal symptoms may become a kind of stress to induce depression. But this still requires more rigorous experimentation to clarify the connection.

Perimenopausal depression is accompanied by a series of clinical symptoms, such as insomnia, loss of appetite, decreased cognition and unusual emotions. Among them, cognition is the main factor. The decline in cognitive function is related to the fluctuation of hormones, and cognition is mainly affected by the decline in estrogen levels (33, 34) and the increase in FSH and LH (35–37). Hormones, as enabling factors, may affect cognitive function by affecting neuroplasticity in the brain (30, 37). In second-generation cognitive theory, environment, physiology, and emotion are considered to be factors affecting cognitive function, and this cognition is embodied (38). The dominant factor in perimenopausal depression is cognitive function, which is affected by embodied experience. Changes in hormones, clinical symptoms, and quality of life all affect cognitive function, and this impact on cognition will ultimately dominate the occurrence of and changes in depression (39).

In this study, escitalopram (an SSRI) and electroacupuncture were used as interventions and compared. The escitalopram group received SSRI antidepressants, which directly affect the reuptake of the neurotransmitter serotonin in the brain and affect the central nervous system (40). In the later period of treatment, the electroacupuncture group showed a gradual decline in MENQOL and HAMD-17 scores. There was no significant difference in hormone levels between the two groups. The efficacy of acupuncture in improving physical symptoms, quality of life, and depression in the menopausal transition period has been confirmed by several studies (41). In this study, the LPA of the EA and MC groups at different times in the treatment process showed that the cognitive dimension was the main dimension that dominated the severity of the HAMD-17 score. With the extension of treatment, the HAMD-17 scores of the two groups showed a gradual decline, and this trend was dominated by changes in cognitive impairment. From the perspective of embodied cognition, clinical symptoms, quality of life, and hormones will all affect cognitive function, and abnormalities in cognitive function will in turn affect symptoms and depression. This may also be one of the reasons why antidepressants that target neurotransmitters can improve cognitive function, quality of life and clinical symptoms when improving depression. Electroacupuncture is a therapy with strong somatosensory properties. In patients with perimenopausal depression, electroacupuncture may not only improve the functioning of the nervous system but also regulate the clinical symptoms. Electroacupuncture may change cognitive function by improving the individual's embodied experience.

This study suggests that hormonal shocks can cause more pronounced perimenopausal symptoms, which makes it easier to induce depression. In clinical practice, for patients with obvious perimenopausal depression, more attention should be paid to the patient's mental state. In the treatment of perimenopausal depression, electroacupuncture and other strong somatosensory therapies can be added as auxiliary therapies. This study provides a new understanding of perimenopausal depression. Future research can explore the role of acupuncture in perimenopausal depression from the perspective of molecular biology, explore the physiological basis of the effectiveness of acupuncture in the treatment of perimenopausal depression.

There are still some limitations in this study, such as the missing estradiol data in hormone collection, insufficient evaluation times, and lack of specific evaluation methods for cognitive function. For the control group, this study selected an SSRI, and in the future, we can consider adding hormone replacement therapy for comparison.

In the future, we can consider adding hormone replacement therapy for comparison and using specific evaluation methods for cognitive function. For perimenopausal depression, a cognitively oriented and embodied affected disease, we can consider adding embodied therapies for intervention, such as yoga, acupuncture, cognitive behavioral therapy, and sports.

## Conclusion

Hormonal shock is more likely induce perimenopausal depression when they cause clinical symptoms and poor quality of life. Cognitive impairment is the main manifestation factor in perimenopausal depression. Hormones, symptoms, and quality of life as a physical experience all affect one's cognitive function, and changes in cognitive function may lead to depression. In treatment, electroacupuncture has a strong somatosensory effect, which may affect cognitive function through embodied experience.

## Data Availability Statement

The data analyzed in this study is subject to the following licenses/restrictions: The data that support the findings of this study are not publicly available due to their containing information that could compromise the privacy of research participants, but are available after Wen-Bin Fu agrees to authorize. Requests to access these datasets should be directed to Jun-He Zhou, zhoujunhe0201@163.com.

## Ethics Statement

The studies involving human participants were reviewed and approved by the Institutional Ethics Committee of GUANGDONG Provincial Hospital of Traditional Chinese Medicine provided ethics approval (B2014-008-01). The patients/participants provided their written informed consent to participate in this study.

## Author Contributions

J-HZ was responsible for data analysis and writing of this article. D-LZ and B-LN were responsible for making corrections to the paper and guiding the analysis methods. W-BF was in charge of this research. X-JX was responsible for data analysis. LZ and QW were responsible for participant recruitment. L-DY was responsible for data collection. All authors contributed to the article and approved the submitted version.

## Funding

The 12th Five-Year National Science and Technology Pillar Program (2012BA124B01), Key-Area Research and Development Program of Guangdong Province (2020B1111100007), Shenzhen Bao'an Research Center for Acupuncture and Moxibustion (BAZJ2018239), and Sanming Project of Medicine in Shenzhen (SZSM201806077). As the main center of this study, Guangdong Provincial Hospital of Traditional Chinese Medicine greatly helped with the development and implementation of this study.

## Conflict of Interest

W-BF has 3 patents, J-HZ has 1 patent, and none of the patent holders obtain any economic benefits from this article. The remaining authors declare that the research was conducted in the absence of any commercial or financial relationships that could be construed as a potential conflict of interest.

## Publisher's Note

All claims expressed in this article are solely those of the authors and do not necessarily represent those of their affiliated organizations, or those of the publisher, the editors and the reviewers. Any product that may be evaluated in this article, or claim that may be made by its manufacturer, is not guaranteed or endorsed by the publisher.
