# Supplementary material for: The Role of Acupuncture in Hormonal Shock-Induced Cognitive-Related Symptoms in Perimenopausal Depression: A Randomized Clinical Controlled Trial
Source: Front Psychiatry. 2022 Jan 13;12:772523. doi: 10.3389/fpsyt.2021.772523 (PMC8793332; doi:10.3389/fpsyt.2021.772523)
Supplement: Supplementary file 1 [file Table_1.docx]

**LPA model fit results of HAMD-17 scale scores.**

| Group | Times | Classes | AIC | BIC | aBIC | Entropy | LMR, P | BLRT, P |
| --- | --- | --- | --- | --- | --- | --- | --- | --- |
| TA&MC | T1 | 2 | 2364.971 | 2408.607 | 2367.414 | 0.600 | 0.0049 | ＜0.001 |
|  |  | 3 | 2331.880 | 2392.299 | 2335.263 | 0.736 | 0.0789 | ＜0.001 |
|  |  | **4** | **2320.890** | **2398.092** | **2325.212** | **0.755** | **0.6883** | **0.0128******* |
|  |  | 5 | 2309.996 | 2403.981 | 2315.258 | 0.823 | 0.3390 | 0.0659 |
| TA | T1 | 2 | 1204.477 | 1239.345 | 1198.268 | 0.685 | 0.094 | ＜0.001 |
|  |  | **3** | **1190.888** | **1239.167** | **1182.292** | **0.799** | **0.1217** | **＜0.001*** |
|  |  | 4 | 1189.356 | 1251.045 | 1178.372 | 0.786 | 0.8927 | 0.375 |
|  |  |  |  |  |  |  |  |  |
|  | T2 | 2 | 1208.689 | 1243.556 | 1204.48 | 0.917 | 0.004 | ＜0.001 |
|  |  | **3** | **1189.007** | **1237.286** | **1180.411** | **0.806** | **0.2349** | **＜0.001*** |
|  |  | 4 | 1135.758 | 1197.477 | 1124.773 | 0.94 | 0.2437 | 0.05 |
|  |  | 5 | 1126.567 | 1201.666 | 1113.195 | 0.91 | 0.09 | 0.02 |
|  |  | 6 | 1116.721 | 1205.231 | 1100.961 | 0.954 | 0.8089 | 0.0128 |
|  |  | 7 | 1112.046 | 1213.967 | 1093.898 | 0.96 | 0.0344 | 0.1333 |
|  |  |  |  |  |  |  |  |  |
|  | T3 | 2 | 1195.551 | 1230.419 | 1189.343 | 0.817 | 0.0075 | ＜0.001 |
|  |  | **3** | **1047.792** | **1096.07** | **1039.196** | **1** | **0.6446** | **＜0.001*** |
|  |  | 4 | 1042.228 | 1103.918 | 1031.244 | 0.942 | 0.1157 | 0.1034 |
|  |  |  |  |  |  |  |  |  |
|  | T4 | **2** | **1182.908** | **1217.776** | **1176.7** | **0.992** | **0.1687** | **＜0.001*** |
|  |  | 3 | 1181.733 | 1230.012 | 1173.137 | 0.867 | 0.235 | 0.2667 |
|  |  |  |  |  |  |  |  |  |
|  | T5 | **2** | **1139.471** | **1174.339** | **1133.263** | **1** | **0.2463** | **＜0.001*** |
|  |  | 3 | 1135.69 | 1183.968 | 1127.094 | 0.766 | 0.4178 | 0.2667 |
|  |  |  |  |  |  |  |  |  |
|  | T7 | 2 | 1095.943 | 1130.811 | 1089.735 | 1 | 0.2409 | ＜0.001 |
|  |  | **3** | **1086.077** | **1134.355** | **1077.481** | **0.77** | **0.4156** | **＜0.001*** |
|  |  | 4 | 1092.336 | 1154.025 | 1081.352 | 0.814 | 0.7143 | 1 |
| MC | T1 | 2 | 1178.941 | 1213.318 | 1172.251 | 0.602 | 0.0576 | ＜0.001 |
|  |  | 3 | 1166.073 | 1213.672 | 1156.81 | 0.691 | 0.6731 | ＜0.001 |
|  |  | 4 | 1150.159 | 1210.98 | 1138.323 | 0.799 | 0.0409 | ＜0.001 |
|  |  | **5** | **917.837** | **991.88** | **903.428** | **1** | **0.683** | **＜0.001*** |
|  |  | 6 | 1138.34 | 1225.605 | 1121.358 | 0.903 | 0.0832 | 1 |
|  |  |  |  |  |  |  |  |  |
|  | T2 | 2 | 1172.415 | 1306.792 | 1165.725 | 0.608 | 0.1373 | ＜0.001 |
|  |  | **3** | **1158.294** | **1205.893** | **1149.031** | **0.763** | **0.22** | **＜0.001*** |
|  |  | 4 | 1145.451 | 1206.272 | 1133.614 | 0.844 | 0.1231 | 1 |
|  |  |  |  |  |  |  |  |  |
|  | T3 | 2 | 1157.907 | 1192.284 | 1151.217 | 0.934 | 0.4852 | ＜0.001 |
|  |  | **3** | **955.000** | **1003.318** | **946.456** | **1** | **0.7632** | **＜0.001** |
|  |  | 4 | 951.92 | 1012.741 | 940.084 | 0.894 | 0.2353 | 0.15 |
|  |  |  |  |  |  |  |  |  |
|  | T4 | 2 | 1142.788 | 1177.165 | 1136.098 | 0.853 | 0.0204 | ＜0.001 |
|  |  | **3** | **947.047** | **994.646** | **937.784** | **1** | **0.777** | **＜0.001*** |
|  |  | 4 | 944 | 1005 | 933.078 | 0.985 | 0.659 | 0.2667 |
|  |  |  |  |  |  |  |  |  |
|  | T5 | 2 | 1127.643 | 1162.02 | 1120.953 | 0.893 | 0.0299 | ＜0.001 |
|  |  | 3 | 933.254 | 980.853 | 923.991 | 1 | 0.7766 | ＜0.001 |
|  |  | 4 | 922.362 | 983.183 | 910.526 | 0.876 | 0.3781 | ＜0.001 |
|  |  | **5** | **901.308** | **975.351** | **886.899** | **0.888** | **0.3687** | **＜0.001*** |
|  |  | 6 | 895.061 | 982.326 | 878.079 | 0.905 | 0.2202 | 0.1111 |
|  |  |  |  |  |  |  |  |  |
|  | T7 | 2 | 1090.258 | 1124.635 | 1083.568 | 1 | 0.2096 | ＜0.001 |
|  |  | **3** | **1057.703** | **1105.302** | **1048.44** | **0.857** | **0.0388** | **＜0.001*** |
|  |  | 4 | 1053.105 | 1113.926 | 1041.269 | 0.861 | 0.8047 | 0.1111 |

***: Selected LPA classification.**

**Code used by Mplus software**

**LPA code**

DATA:

FILE IS E:\***.dat;

VARIABLE:

NAMES ARE VAR1 VAR2 VAR3 VAR4 VAR5;

USEVARIABLES ARE VAR1 VAR2 VAR3 VAR4 VAR5;

CLASSES = c (n);

ANALYSIS:TYPE=MIXTURE;

STARTS=200 50;

OUTPUT: TECH11 TECH14;

SAVEDATA:

FILE=LPA.txt;

save=cprob;

Plot:

type is plot3;

series= VAR1 VAR2 VAR3 VAR4 VAR5;

**Structural equation model code**

DATA:

FILE IS **.dat;

VARIABLE:

MISSING ARE ALL (-99);

NAMES ARE VAR1 VAR2 VAR3 VAR4 VAR5 VAR6 VAR7 VAR8 VAR9 VAR10 VAR11;

USEVARIABLES ARE VAR1 VAR2 VAR3 VAR4 VAR5 VAR6 VAR7 VAR8 VAR9 VAR10 VAR11;

ANALYSIS:

TYPE IS GENERAL;

ESTIMATOR IS ML;

BOOTSTRAP=1000;

MODEL:

Hor BY VAR1 VAR2;

MENQOL BY VAR3 VAR4 VAR5 VAR6;

HAMD BY VAR7 VAR8 VAR9 VAR10 VAR11;

MENQOL ON Hor;

HAMD ON Hor MENQOL;

MODEL INDIRECT:

HAMD IND Hor;

OUTPUT:SAMPSTAT STDYX CINTERVAL(BOOTSTRAP);
