# Supplementary material for: The Role of Acupuncture in Hormonal Shock-Induced Cognitive-Related Symptoms in Perimenopausal Depression: A Randomized Clinical Controlled Trial
Source: Front Psychiatry. 2022 Jan 13;12:772523. doi: 10.3389/fpsyt.2021.772523 (PMC8793332; doi:10.3389/fpsyt.2021.772523)
Supplement: Supplementary file 2 [file Data_Sheet_2.doc]

**NO.2012BAI24B01**

**National Science and Technology Support Plan for the 12th Five-Year Plan**

**Project Title: International multi-center clinical evaluation study on the efficacy of acupuncture and moxibustion**

**Effects and safety of electroacupuncture in mild to moderate depression in perimenopausal period**

**-A Multi-center randomized controlled study**

**Protocol**

**VERSION1.0_20131201**

**Contents**

[1. Introduction 3](#__RefHeading___Toc75425604)

[2. Study design 4](#__RefHeading___Toc75425605)

[2.1 Design and sample size 4](#__RefHeading___Toc75425606)

[2.2 Participants 6](#__RefHeading___Toc75425607)

[2.2.1 Diagnostic criteria 6](#__RefHeading___Toc75425608)

[2.2.3 Exclusion criteria 7](#__RefHeading___Toc75425609)

[2.3 Study process 7](#__RefHeading___Toc75425610)

[2.4 Intervention 8](#__RefHeading___Toc75425611)

[2.5 Outcomes 9](#__RefHeading___Toc75425612)

[2.6 Adverse events 10](#__RefHeading___Toc75425613)

[2.7 Data management 11](#__RefHeading___Toc75425614)

[2.7.1 Original data management and archiving 11](#__RefHeading___Toc75425615)

[2.7.2 Data Entry and Storage 11](#__RefHeading___Toc75425616)

[2.8 Statistical Analysis 12](#__RefHeading___Toc75425617)

[2.8.1 ITT analysis 12](#__RefHeading___Toc75425618)

[2.8.2 Contents and methods of statistical analysis 12](#__RefHeading___Toc75425619)

[2.8.3 Other statistical analysis 12](#__RefHeading___Toc75425620)

[2.9 Ethics 13](#__RefHeading___Toc75425621)

# 1. Introduction

Perimenopausal syndrome seriously affects women's health and life. Due to the decline of reproductive function, perimenopausal women have a series of physiological changes, which are manifested as a series of clinical symptoms such as hot flashes, insomnia, pain, depression, and fatigue. Perimenopausal depression refers to depression that first occurred during perimenopause, with anxiety, depression as the main symptoms.

Studies have shown that women are at greater risk of depression during the perimenopausal period, and there is a correlation between the perimenopausal period and the occurrence of depression. At present, there are still controversies regarding the treatment of perimenopausal depression. Most clinical studies believe that depression is the main cause of the disease, and antidepressants are mainly used for treatment, and someone have proposed antidepressants and hormones. The combined application of alternative therapies can enhance the antidepressant effects of drugs. Regarding the treatment of perimenopausal depression with acupuncture, studies shows that acupuncture can improve various physical discomforts and negative emotions of perimenopausal women, reduce the patient's HAMD score and SDS score. Previous clinical studies on acupuncture and moxibustion for treatment of perimenopausal depression have many shortcomings, and the study quality is not high. Therefore, this project is based on the above problems, starting with the study of electroacupuncture intervention in perimenopausal mild to moderate depressive disorder, through a large sample multi-center RCT trial study with reasonable design, standard operation and follow-up after treatment to reveal the efficacy and specificity of acupuncture in the treatment of perimenopausal depression.

This study intends to answer the question: Is acupuncture effective in perimenopausal mild to moderate depression?

# 2. Study design

## 2.1 Design and sample size

This study is a multi-center randomized controlled study, with 6 centers. Using central randomization, the electroacupuncture group (EA group) and the drug group (MC group) were set up, and the sample size of the two groups was allocated at a ratio of 1:1. The common technology platform established by the subject "Research on Common Technology and Data Management of Acupuncture Clinical Efficacy Evaluation" is implemented in a unified manner.

**Randomization method** The participants are randomly assigned to the electroacupuncture group and the drug group according to a ratio of 1:1. The "Proc plan" program of SAS9.3 statistical analysis software was used to generate the random plan. When qualified participants are enrolled in the group, the randomization or clinical researchers in each center log in to the central randomization system via telephone or the Internet to apply for a random number. The randomization plan of this study was produced by the Clinical Evaluation Center of the Clinical Institute of China Academy of Chinese Medical Sciences, and relevant researcher did not participate in the statistical analysis of this study. The central random system has set strict personnel permissions, except for the highest-level system administrators, no one else has the right to view the random plans in the central random system.

**Control** Based on published articles on the treatment of perimenopausal depression, this trial used escitalopram as a control. The patient took 10 mg daily for 12 weeks.

**Blinding** This study blinds participants, curative effect evaluators and statistical analysts. During the entire trial, only the acupuncture operator knew the grouping of patients. This study uses the method of making appointments for patients to ensure that there is no contact and communication between the two groups of patients, and the patients should be blinded when the medicine is recovered and counted.

**Sample size** The non-inferiority test adopts a one-sided inspection level α. Assuming that the allowable probability of the second type of error does not exceed β, the population rate of the two groups is the same, that is, under the condition of T=S=P.


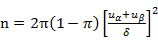


In this study, the main curative effect index is the HAMD-17 scale score, and the evaluation method in the curative effect index in the study is: HAMD-17 score reduction rate ≥50% is significantly effective; HAMD-17 score reduction rate ≥25%, <50% is effective ; HAMD-17 score reduction rate <25% is invalid. Related studies showed that the reduction rate of acupuncture treatment and drug treatment is about 80%, take T=S=π=0.8, according to level of α=0.05, β=0.10, the u 0.05=1.645, u 0.10=1.282, the maximum δ value does not exceed 1/5 of the control group rate. According to this calculation, the maximum δ=0.16, and substituting into the formula to calculate n=107, that is, if the clinical effectiveness of the two programs is 80%, α =0.05 as a significance level, each group of 107 patients participating in the test will have a 90% confidence in obtaining a non-inferior conclusion that the curative effect of acupuncture is not worse than the drug. Combined with clinical practice, the maximum dropout rate is 15%, so the actual maximum number of cases required for each group is n=107*1/(1-0.15)≈126 cases, and a total of 252 cases are required in the two groups.

## 2.2 Participants

### 2.2.1 Diagnostic criteria

Diagnostic criteria

Depressive disorder that first occurred during perimenopause meets the perimenopausal diagnostic criteria updated by the American Society of Reproductive Society (STRAW-10) in 2012, which includes the transitional period of menopause and one year after menopause. At the same time, it meets the American Psychiatric Association DSM-5 diagnostic criteria for the onset of depression caused by other physical conditions, and the degree is in line with the International Classification of Diseases (ICD-10) mild depression (F32.0) or moderate depression (F32) .1), or HAMD-17 score <23 points, ≥8 points.

Perimenopausal standards:

Refers to the STRAW-10 of Menopausal Transition Standard. ( Figure 1)。

***Figure 1***

**2.2.2** **Inclusion criteria**

Perimenopausal depression participants (aged 45-55 years, 8≤HAMD＜23) were eligible for enrollment if they conformed to the criteria in STRAW-10 and DSM-5 and had not used any hormone replacement therapy or antidepressant drugs during the last three months. The participants were experiencing first-onset depressive disorder while agreeing to participate in the study and signing the informed consent form.

### 2.2.3 Exclusion criteria

Exclusion criteria included allergy to escitalopram and other drugs that may cause drug-to-drug interactions. Patients with ovarian cysts, uterine fibroids larger than 4 cm in diameter, ovaries or after hysterectomy, SCL-90 score failed to rule out suicidal tendencies (>26), and diseases considered by the investigator to be inappropriate for participating in this study will be excluded.

## 2.3 Study process

The study is from October 10, 2013 to October 31, 2015. The last participant was included on April 30, 2015, and the deadline for case observation was October 30, 2015. The study period for all participants was 24 weeks, the treatment intervention period after enrollment was 12 weeks, and the follow-up period was 13-24 weeks. Assessment was carried out at 0, 4, 8, 12, 16, 20, and 24 weeks.

## 2.4 Intervention

Needle: Huatuo brand disposable tube needle φ0.30×25mm (1 inch), φ0.30×40mm (1.5 inch), φ0.30×50mm (2 inch), φ0.30×75mm (3 inch); Electroacupuncture instrument: SDZ-V Huatuo brand electronic acupuncture instrument. Needles and electroacupuncture were purchased from Suzhou Medical Supplies Factory Co., Ltd. (Production Enterprise License: Su Food and Drug Administration Apparatus Production License No. 2001-0020; Registration Certificate: Su Food and Drug Administration Apparatus (quasi) 2004 No. 2270202).

**Electroacupuncture group (EA)**

The acupoints are GUANYUAN, ZIGONG, TIANSHU, SANYINJIAO, HEGU, TAICHONG (both selected acupoints on both sides), BAIHUI, YINTANG. Acupoints position refer to the 2006 National Standard of the People’s Republic of China (GB/T 12346-2006) "Acupoint Names and Positioning". Participants in the acupuncture group underwent a connected 50 Hz, 0.5-10 mA current stimulation between the needle handles of BAIHUI and YINTANG, bilateral ZIGONG and bilateral TIANSHU, and each treatment occurred three times per week for 30 minutes, with an interval of more than 24 hours.

**Escitalopram group (MC)**

Orally take 10 mg of escitalopram oxalate at 0.5h every day after breakfast for 12 weeks of treatment. Escitalopram Tablets (Lexapro), Denmark H. Lundbeck A/S, Escitalopram Oxalate Tablets (Lexapro), registration number: H20100541, 2010-07-29, 10mg.

During the study period, it is not allowed to use other specialized treatment drugs for alleviating perimenopausal depression. If the drugs have been used, they are considered to be excluded and should be recorded in detail in the "drug use record form" and the "one week drug use" in the CRF table.

## 2.5 Outcomes

**Primary outcome**

The depression of the participants was assessed by the HAMD-17 scale which has five dimensions including somatization, weight, cognitive impairment, block, and sleep. The assessment of HAMD-17 scale was collected at 0, 4, 8, 12, 16 and 24 weeks.

**Secondary outcome**

MENQOL and serum E_2_, FSH, LH. MENQOL evaluation times at 0, 4, 8, 12, 16, 20, 24 weeks. E_2_, FSH, and LH evaluation times at 0 and 12 weeks. Hormones were collected on 2-5 days during the menstrual cycle in the 0^th^ and 12^th^ weeks of the treatment period, and FSH, LH, and E_2_ were taken from venous blood at 8-9 am on an empty stomach.

**Safety outcome**

Blood test, electrocardiogram, liver function (AST/ALT/TBIL), renal function (BUN/Cr).

**Demographic data**

Including BMI, race, marital status, working status, allergy history, menopause stages.

## 2.6 Adverse events

**Dizziness**

Patients receiving acupuncture treatment for the first time should carefully explain the acupuncture procedures and the resulting sensations to the patient before the start of the treatment, closely observe the patient's complexion and pulse, and detect any pre-symptoms of fainting needles. Once symptoms appear, the needle should be immediately taken out and the patient should lie supine. In severe cases, first-aid measures should be given. If the symptoms persist, necessary first-aid measures should be taken.

**Pain**

Pain during needle insertion is usually caused by the operator's unskilled needle insertion technique, or the needle tip is blunt, hooked. Sometimes it also occurs in overly sensitive participants. The needle should be gently twisted back and forth to loosen the entangled fibers and relieve pain. The pain when the needle is retained is usually caused by the patient's movement, and it needs to be restored to the original position to relieve it.

If the investigator finds that a participant has an adverse event/reaction caused by acupuncture, it should be recorded in the "adverse event record sheet" in the CRF, and adequate comfort and treatment should be given to the patient. If there is a serious adverse event, it should be reported to the Second Affiliated Hospital of Guangzhou University of Traditional Chinese Medicine immediately. Contact: Wen-bin Fu 13808888626.

## 2.7 Data management

### 2.7.1 Original data management and archiving

This study uses Remote Data Capture (RDC) system for data entry. All CRFs are directly entered into the RDC system by the researcher, and all electronic CRFs take effect after being reviewed by the researcher and approved by the electronic signature. All CRF and CRF revision traces are kept in the Oracle database.

### 2.7.2 Data Entry and Storage

Perform CRF annotations and write CRF annotation plans for CRF in accordance with the principles of CDISC-SDTM, while use Oracle Clinical software to create a data entry interface. The data entry interface is as consistent with the CRF as possible, and the entered data is stored in the Oracle database. The data generated in this study will be double entered by two data entry clerks. A data entry guide will be prepared based on the research content, and special entry training will be given to data entry staff before data entry. The data entry clerk performs data entry according to the entry guide. It will take effect after being reviewed and approved by the researcher and electronically signed. The content of the code is medical history, adverse events and concomitant medications. The medical history and adverse events will be coded according to the MedDRA dictionary (Version 13.0).

## 2.8 Statistical Analysis

### 2.8.1 ITT analysis

All randomized participants constitute the randomized population of this study. According to ITT principles, statistical analysis will be performed on all randomized participants.。

### 2.8.2 Contents and methods of statistical analysis

All statistical tests used two-sided tests. A *P* value of less than 0.05 would be considered as statistically significant. The measurement data are described by the mean ± standard deviation, and the count data and grade data are described by the number of cases and percentages. The comparison between groups will be based on the data distribution characteristics to choose the analysis method: measurement data use group t test or non-parametric test, count data use χ2 test or Fisher exact probability method, grade data use non-parametric test.

### 2.8.3 Other statistical analysis

***Structural equation model***

The variables FSH, LH, and E_2_ were defined as latent variables for hormones, the 5 dimensions of the HAMD-17 were defined as latent variables for depression, and the 4 dimensions of MENQOL were defined as latent variables for perimenopausal symptoms and quality of life. Pearson and Spearman correlation analyses were used to test the relationship between the dimensions of latent variables. We used a structural equations model (SEM) to test the mediation relationship among latent variables. The structural equation model was conducted by using MPLUS (version 8.0). The model fit was assessed with the comparative fit index (CFI, critical value≥0.9), the Tucker Lewis Index (TLI, critical value≥0.9), the root mean square error approximation (RMSEA, value blew 0.08), and the chi-square divided by degrees of freedom < 3 (χ^2/df^).

***Latent profile analysis***

Latent profile analysis was used to distinguish the characteristics of different dimensions on the HAMD-17 scale. The MPLUS could complete the LPA in HAMD-17 scale. Each latent profile analysis was analyzed from a 2-class model, and the number of classes was gradually increased until the optimal model was fitted. Model fit was assessed from three indices: information criteria (CI), entropy and Lo-Mendell-Rubin (LMR). Information criteria consisted of the Akaike information criterion (AIC), Bayesian information criterion (BIC) and adjusted Bayesian information criterion (aBIC). A better model fit included the smallest information criteria, entropy approaching 1, and Lo-Mendell-Rubin *P*＜0.05.

## 2.9 Ethics

This study was conducted in accordance with the ethical principles stated in the Declaration of Helsinki (2000 edition) and the relevant clinical research norms and regulations in China. The study must pass the review of the ethics committee of each center research unit before clinical research can be started.
